# Effect of time restricted feeding on anthropometric measures, eating behavior, stress, and brain-derived neurotrophic factor (BDNF) and lipopolysaccharide-binding protein (LBP) levels in women with overweight/obesity and food addiction: a study protocol for a randomized clinical trial

**DOI:** 10.1186/s13063-022-06439-x

**Published:** 2022-06-10

**Authors:** Hanieh Irani, Banafsheh Khodami, Behnaz Abiri, Atoosa Saidpour

**Affiliations:** 1 Department of Nutrition, Faculty of Nutrition and Food Sciences, Shahid Beheshti University of Medical Sciences, Tehran, Iran; 2 Obesity Research Center, Research Institute for Endocrine Sciences, Shahid Beheshti University of Medical Sciences, Tehran, Iran

**Keywords:** Food addiction, Time-restricted feeding, Intermittent fasting, BDNF, LBP, Eating behavior, Weight loss, Gut microbiome

## Abstract

**Background:**

Food addiction is one of the behavioral factors that play an important role in the pathogenesis of obesity. Much evidence is available suggesting intestinal microbiomes can play a role in eating behavior, body composition, and BDNF levels, and they can be modified by time-restricted feeding (TRF). So, this study will aim to evaluate the effect of TRF on anthropometric measures, eating behavior, stress, and serum BDNF and LBP levels in women with overweight/obesity and food addiction.

**Methods:**

We will carry out a randomized clinical trial for 8 weeks to evaluate the effect of a TRF on anthropometric measures, eating behavior, stress level, serum BDNF and LBP levels in women with overweight/obesity and food addiction.

**Discussion:**

Given the effect of BDNF on regulating eating behavior and body weight and the effect of dietary restrictions on BDNF and the gut microbiome, the TRF diet could possibly be a new way to successfully manage weight through modifying BDNF in people with eating disorders, including food addiction.

**Trial registration:**

Iranian Registry of Clinical Trials IRCT20131228015968N7. Registered on 25 October 2020.

**Supplementary Information:**

The online version contains supplementary material available at 10.1186/s13063-022-06439-x.

## Background

Despite recent advances in our understanding of the physiological mechanisms that regulate body weight, obesity remains a major health problem worldwide with multiple consequences, including metabolic and endocrine complications, malignancy diseases, and psychosocial problems [1]. The global obesity epidemic suggests that obesity is not only triggered by a lack of motivation for weight loss, loss of control over food intake, and continued excessive consumption despite knowing negative consequences may also develop in many individuals [2]. The term of “food addiction” is used to describe these compulsive feeding behaviors associated with loss of control of eating [3, 4] with a prevalence rate ranging from 19 to 56.8% in different populations [4, 5]. The prevalence of food addiction among Iranian women with obesity was 26.2% based on the Iranian version of the Yale Food Addiction Scale [5]. Feeding behavior can be managed by both homeostatic (associated with energy demands/stores) and hedonic pathways (brain dopaminergic reward system) which controls energy intake and body weight [6]. So, understanding the mechanisms underlying feeding behavior might be helpful in finding the way of being more efficient in obesity management. Some appetite-regulating hormones such as brain-derived neurotropic factor (BDNF) have been shown to play a modulatory role in reward-related behaviors through both aforementioned pathways [7, 8]. So, it seems that modification in eating behavior through modulation of these hormones may play a key role in some cases of weight gain and obesity such as food addicted ones. Peripheral and central levels of BDNF are associated and BDNF can cross the blood-brain barrier in both directions [9]. Gut microbiota modulation is one of the new therapeutic approaches for managing feeding behavior [10]. Based on previous studies, gut microbiota may play an important role in regulating obesity, energy balance, and also the host eating behavior through affecting appetite and hormone levels such as BDNF [10, 11]. BDNF is a neuromodulator playing an important role in the homeostatic control of food intake and energy expenditure regulation [8]. The regulatory role of BDNF in hedonic feeding has also documented through the mesolimbic reward pathways [8] including consumption of palatable food. So it is not surprising that disruptions in these aforementioned regulatory roles of BDNF lead to hyperphagic behavior and obesity [12]. So far, it seems that gut microbiota improvement might have a modulatory effect on central levels of BDNF [13–16].

Several methods have been suggested to modify and improve the gut microbiota such as probiotic and prebiotic supplementation and fasting [17, 18]. Time-restricted feeding (TRF) is a kind of fasting recommending individuals to confine the eating window to a specified number of hours per day, without altering calorie intake or diet quality [19]. TRF could alter the diversity or abundance of the gut microbiome in response to the body’s circadian rhythm [20]. In turn, gut microbiota can modulate BDNF expression in the brain [21] and also feeding behavior [10]. Feeding behavior can be affected by stress level in individuals and a significant correlation between food addiction and stress has been reported in individuals with overweight and obesity [22, 23]. Lipopolysaccharide (LPS) is an endotoxin produced by altered gut microbiota and is associated with low-grade inflammation in obesity [24, 25]. Lipopolysaccharide-binding protein (LBP) is an acute phase protein produced by the liver which binds to LPS and is a part of LPS-induced inflammation. Serum LBP can be measured to evaluate changes in the gut microbiome [24, 25]. On the other hand, previous clinical trials documented that TRF might have a favorable effect on weight loss, reduction of insulin resistance, systolic blood pressure, and fasting glucose levels [26]. However, some previous studies did not show any advantages of using TRF on body weight management [27, 28]. Therefore, these aforementioned discrepancies may be explainable by ignoring the role of feeding behavior disorders such as food addiction on body weight management. Hence, the aim of this clinical trial is to investigate the effect of TRF on anthropometric indices, stress level, eating behavior, and serum levels of BDNF and LBP in women with overweight/obesity and food addiction.

### Objectives and hypothesis

Primary aims of this clinical trial are the changes in anthropometric indices (including weight, BMI, hip circumference, waist-to-hip ratio, fat mass, muscle mass) and food addiction score. Secondary aims are changes in stress level, eating behavior, and serum levels of BDNF and LBP. A TRF is expected to improve these aforementioned indicators.

## Methods and design

### Study design

**Fig. 1:**
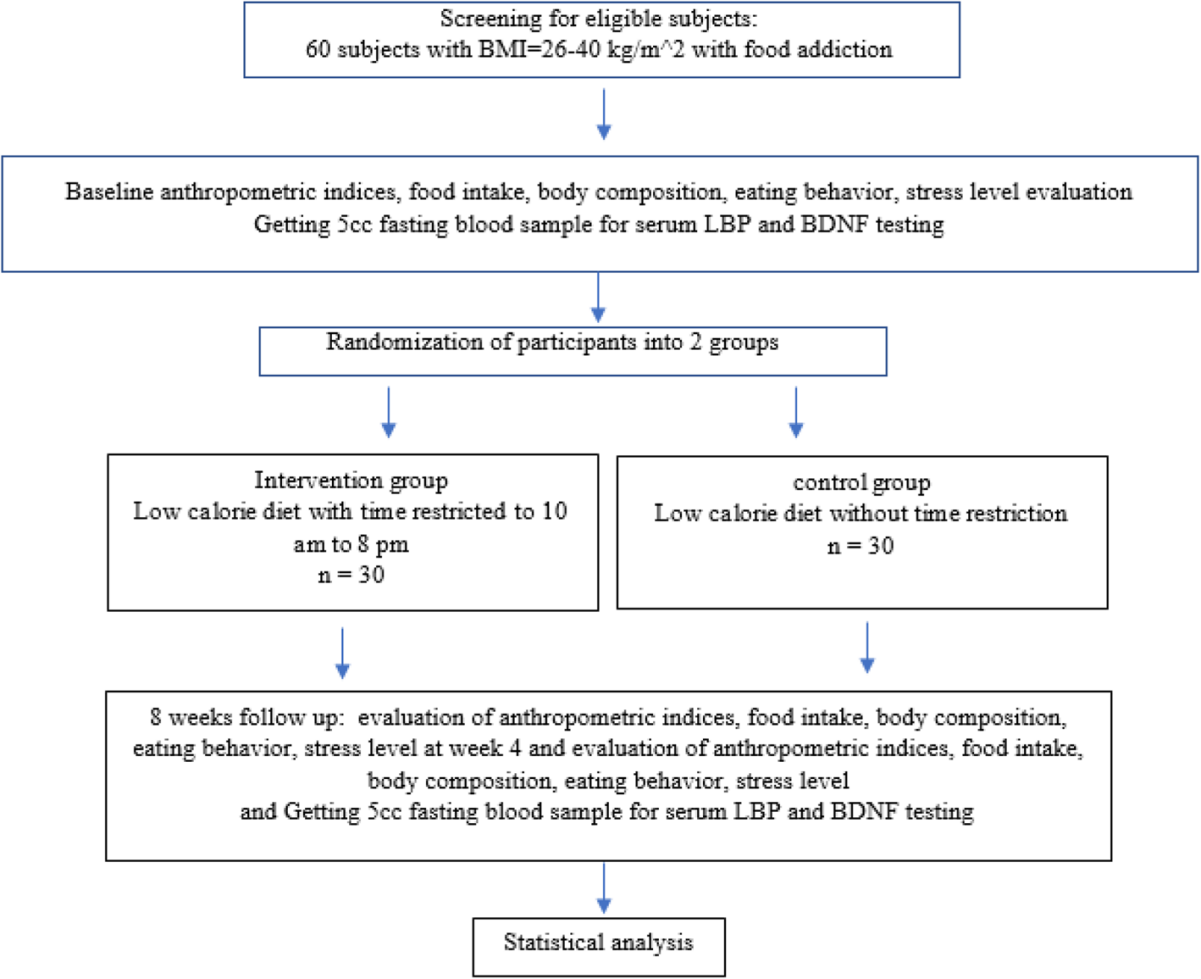
Protocol flow diagram; we will carry out an 8-week randomized controlled trial to determine the effects of a time-restricted feeding on anthropometric indices, body composition, eating behavior, stress, and brain-derived neurotrophic factor (BDNF) and lipopolysaccharide-binding protein (LBP) levels in overweight and obese women with food addiction

**Fig. 2:**
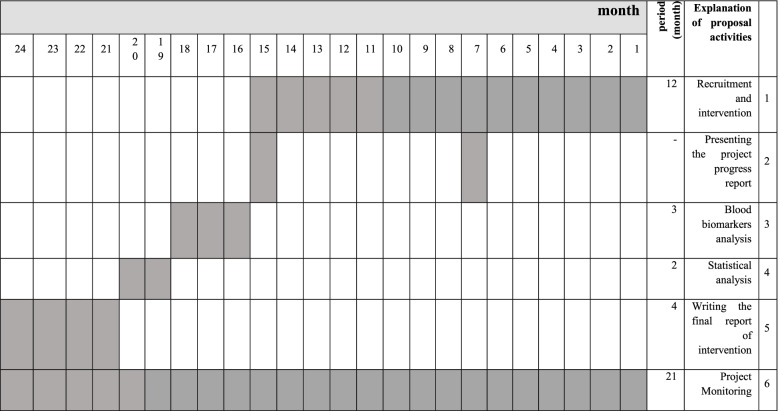
Timeline of the study; we expect the duration of the trial will be 24 months

We will carry out an 8-week double-blind randomized controlled trial. The flow chart of the study is presented in Fig. 1. This intervention will be conducted in the nutrition clinic of Shahid Beheshti University of Medical Sciences in Tehran, Iran, to evaluate the effect of TRF on anthropometric indices, eating behavior, stress, serum BDNF and LBP levels in women with overweight/obesity, and food addiction (Fig. 2).

The protocol is written in line with the Standard Protocol Items: Recommendations for Interventional Trials (SPIRIT) checklist (Additional file 1).

### Sample size

The number of participants was calculated based on the changes in body weight, as a primary outcome. The number of participants in this study was determined by the difference in weight loss between the TRF and control groups of 2.3 kg [28]. This difference has a probability of 95% (*α*=0.05) and a power of 80% (*β*=20%), which is statistically significant. Using this method, each group was estimated to have 26 participants, so that after taking into consideration 20% attrition, every group will be comprised of 30 participants. Collectively, 60 women with overweight or obesity and food addiction will be enrolled.

### Study population

Women with overweight or obesity (BMI: 26– 39.9 kg/m^2^) and with food addiction will be recruited from nutrition clinic of Shahid Beheshti University of Medical Sciences (SBMU) through advertisement and face-to-face communication. The diagnosis of food addiction will be based on the YALE Food Addiction Scale [29]. Women with overweight or obesity that obtain the necessary score will be enrolled in the study. Subjects who meet the inclusion criteria will be completely informed about the protocol of the study. Each participant will sign the informed consent form.

### Inclusion and exclusion criteria

For the present study, 60 adult women with the following inclusion criteria will be included: aged 20–65 years; BMI: 26–39.9 kg/m^2^; willingness to participate in the study; confirmation of food addiction after obtaining the necessary score (at least 1 clinical score+ at least having 3 out of 7 criteria) from the YALE food addiction questionnaire; not having any diseases such as cancer, diabetes, newly hypothyroidism, renal or liver failure; non-pregnant or lactating; not perimenopause (women with irregular menstruation due to menopause or less than 2 years after amenorrhea); not participating in a weight loss diet during the last 2 months; and were not smokers.

Patients will be excluded if taking any antibiotics in the last 1 month or during the study; taking any medication affecting study outcomes regularly; using probiotic products (probiotic supplements, yogurt, cheese, cakes, biscuits, and probiotic pasta) continuously (more than once a week) in the last month or during the study; using weight-loss or appetite-suppressing medications; drinking alcohol at any levels before or during the study; and women with irregular menstruations. Also, we will exclude participants who refuse to continue the study, need antibiotics or have difficulties in fasting for 14 h a day in the intervention group.

### Randomization, sequence generation, and concealment

Participants will be randomly allocated into two groups: the group receiving a low-calorie diet with the time-restricted feeding protocol or the control group receiving a low-calorie diet and will be followed up for 8 weeks. Stratified blocked randomization method is used to randomly assign people to two groups. The sequencing will be generated by one of the authors (AS) who is not going to assign participants into the study, so, allocation concealment will be ensured, as the author will not release the randomization code until the patient has been recruited into the trial by other authors (HI and BKh), which takes place after all baseline measurements have been completed. Participants are classified into overweight (26–29.9 kg/m^2^), grade 1 (30–34.9 kg/m^2^), and grade 2 obesity (35–39.9 kg/m^2^) based on BMI and randomly assigned to one of the control or TRF groups. Separate randomization is performed within each group for each BMI class. The size of the blocks is 4, two allocations are given to TRF group (A) and two allocations to Control Group (B). Six different permutations of AABB, ABAB, BBAA, BABA, ABBA, and BAAB will be created.

### Implementation

Potential participants will be invited to the study by HI and BKh. The sequencing will be generated by AS, who is not going to assign participants into the study.

### Intervention

In this study, patients receive the relevant diets for 8 weeks based on the group they are placed in. In both groups, after calculating the amount of calories required by each individual using the Mifflin formula [30] as much as 300–500 kcal will be deducted from the total energy required for each person (a 500 kcal reduction from calories is using for most of the cases, except for whom the calorie falls below 1200 kcal/d with 500 kcal reduction. In these cases, 300–400 kcal reduction will be used.) and accordingly, the low-calorie diet plan is given to each individual. The diet in each group will consist of 50–55% carbohydrates, 15–20% protein, and 30% fat. Patients in the TRF group will receive their meals from 10 h a day from 10 am to 8 pm [31] while the control group will have less than 12 h fasting.

### Adherence

In order to control the participants in terms of adherence to the regimen and prevent the loss of samples, they will be followed up twice a week by phone call. At every phone call we will make sure if the cases are still in the study and they do not have exclusion criteria (any need for antibiotic consumption, consuming probiotics, and…). Also, a 24-h recall of the last day is obtaining from individuals and it is compared to their diet to make sure if they are adhering to the diet. The data of the patients with more than 90% compliance with the intervention will be analyzed.

### Study outcomes

Primary outcomes of this clinical trial are the changes in anthropometric indices (including weight, BMI, hip circumference, waist-to-hip ratio, fat mass, muscle mass) and food addiction score. Secondary outcomes are changes in stress level, eating behavior, and serum levels of BDNF and LBP.

After obtaining the informed consent, the general profile sheet will be completed for each patient. Also, at the beginning of the study, the weight of each patient with light clothing and with the accuracy of 100 g and the height of each patient in a shoeless state will be measured by the meter mounted on the wall with an accuracy of 0.5 cm. Then, BMI of patients will be calculated and waist and hip circumferences are measured using meters with 0.5 cm accuracy. Also, the waist-to-hip ratio will be calculated. Then, body fat mass and muscle mass are measured by bioelectrical impedance analysis. The participants’ daily physical activity levels will be measured using the Standard Physical Activity Questionnaire (MET). The validity of this questionnaire has been confirmed [32]. Stress levels of subjects at the beginning and end of the study will be assessed using the Perceived Stress Questionnaire (PSS-14) [33]. The scoring method is that based on the 5-degree spectrum, a score of 0–4 is awarded to each item (never score 0 and most of the time score 4). Phrases 4-5-6-7-10 and 13 are scored inversely (never score 4 to most of the time score 0). Then, by collecting the items, the overall score is 0-56, which the higher score indicates more perceived stress [33]. Eating behavior will be measured using a three-factor eating questionnaire at the beginning and the end of the study. The questionnaire consists of 18 questions in 3 sections about cognitive factors related to eating, hunger, and emotional eating. The questionnaire is scored by questions 1 to 13 on a four-point Likert scale from one (definitely incorrect) to four (definitely correct). Questions 14 to 17 also have a separate Likert scale, and question 18 has an 8-degree Likert scoring scale. The higher rating in the cognitive factors associated with eating indicates a greater limitation in receiving calories to control body weight. Also, a higher hunger score indicates a person’s greater susceptibility to eating in response to hunger and a higher emotional eating score indicates a person’s greater susceptibility to excessive eating. The validity of this questionnaire has been measured in Iran [34].

In order to measure blood biochemical parameters, 5 cc of venous blood samples will be taken from the site of the bracing vein and after 12 to 14 h fasting by the laboratory technician at the beginning and the end of the study. Blood samples taken in tubes containing sodium citrate anticoagulants will be collected and centrifuged in the laboratory of the Shahid Beheshti Nutrition Faculty for 15 minutes at a speed of 500 rounds per minute and serums will be stored at − 80°C until the tests are performed. Serum levels of BDNF and LBP will be measured by the ELISA method with a human BDNF kit (PadginTeb, under the license of Zelbio, Iran) and a human LBP kit (PadginTeb, under the license of Zelbio, Iran) with the intra-assay and inter-assay CV of < 10% and <12%, respectively, for both kits in the laboratory of the Nutrition Institute of Shahid Beheshti University of Medical Sciences.

### Assessment of dietary intake

In this study, to assess participant’s dietary intake, at the beginning of the study, at the end of the fourth and eighth weeks of the study, 3 days of dietary recall about one weekend day and two week days will be completed through face-to-face or telephone interviews. Common household measurement tools (glass, cup, soup bowl, plates, teaspoon, and tablespoon) will be provided to assist subjects in estimating the portion size of the food. Dietary intake will be analyzed using Nutritionist IV (N4) software.

### Assessment of physical activity level

Physical activity questionnaire will be completed for them at baseline and week 8 [32]. This questionnaire is divided into 9 levels based on the intensity of physical activity and metabolic equivalents (MET) and its rows are adjusted from top to bottom from inactivity (MET = 0.9) to intense activities (MET >6). The intensity of activities from top to bottom is 0.9, 1, 1.5, 2, 3, 4, 5, 6, and above 6, respectively. These numbers are multiplied by the duration of the activity to show the intensity of the activity performed per time unit (MET.time).

### Statistical analysis

In this study, data analysis will be performed by using SPSS version 21.0 (SPSS Inc, Chicago, Illinois) software. Paired *t* test will be used to compare the mean of quantitative variables with normal distribution in each group between the beginning and the end of the study and the *t* test will be used to compare their mean between the two groups at the beginning and end of the study. In the case of quantitative variables with non-normal distribution, Wilcoxon and Mann-Whitney tests are used, respectively. In case of the variables measured three times during the study (beginning, fourth week, and eighth week), repeated ANOVA test will be used. Covariance analysis will be used to eliminate the effect of quantitative confounding factors. Quantitative confounding factors are physical activity and baseline values of the biochemical markers. Chi-square test will be used to compare the qualitative variables between the two groups and regression analysis is used to eliminate the effect of qualitative confounding.

### Plans for auditing trial conduct

There will be unplanned checks on the quality of the data or the progress of the trial.

### Ethical considerations

Women with overweight/obesity and food addiction who meet the inclusion criteria will be completely informed about the protocol of the study. The protocol of this study was approved by ethics committee of Shahid Beheshti University of Medical Sciences and is in conformity with the declaration of Helsinki (approved number IR.SBMU.NNFTRI.REC.1399.03).

### Protocol amendments

Any modifications to the protocol which may impact on the conduct of the study, potential benefit of the patient, or may affect patient safety, including changes of study objectives, study design, patient population, sample sizes, study procedures, or significant administrative aspects will require a formal amendment to the protocol. Such amendment will be agreed upon by NNFTRI (National Nutrition and Food Technology Institute) and approved by the ethics committee of Shahid Beheshti University of Medical Sciences prior to implementation. Administrative changes of the protocol are minor corrections and/or clarifications that have no effect on the way the study is to be conducted. These administrative changes will be agreed upon by NNFTRI. The ethics committee of Shahid Beheshti University of Medical Sciences may be notified of administrative changes at the discretion of NNFTRI.

## Discussion

Emerging evidence suggests that intermittent fasting has favorable effects on metabolic health and weight management [19]. A TRF is a kind of intermittent fasting that restricts eating window to less than 12 h per day [19]. It is suggested that TRF helps with reducing body weight, fasting blood sugar, systolic blood pressure, and blood lipids. Some studies suggest that TRF can also alter the gut microbiota [19].

LPS, as an endotoxin and inflammation marker with short half-life, is produced and released to blood circulation by gut microbiome and binds to LBP. High levels of LPS and LBP is representative of altered gut microbiome [24, 25].

Food addiction is one of the behavioral factors that is associated with adiposity and failure in lifestyle changes for weight loss [2–4]. People with higher stress levels are expected to be more susceptible to food addiction [22, 23]. In 2018, Bistoletti et al. suggested that dysbiosis can affect BDNF levels differently in ENS and CNS [35]. Alteration in BDNF levels can be associated with eating behavior and weight management [12] and previous studies on mice showed that compounds produced by gut microbiomes can alter brain levels of BDNF [13–16].

Based on previous studies, it seems that alteration in BDNF levels as a result of alteration in gut microbiota caused by TRF may be a novel strategy for management of food addiction and obesity. So, the aim of this study is to investigate the effect of TRF on anthropometric measures, eating behavior, stress, and serum levels of BDNF and LBP in women with overweight/obesity and food addiction.

## Trial status

This trail is in the enrolment stage.

Protocol version 2, 5/18/22.

Recruitment began on November 2021 and is expected to be completed on December 2023.

## Supplementary Information

**Additional file 1. **SPIRIT Checklist for *Trials*.

## Data Availability

Not applicable.

## Abbreviations

BMI: Body mass index
TRF: Time-restricted feeding
LBP: Lipopolysaccharide-binding protein
BDNF: Brain-derived neurotrophic factor
